# Reactive Oxygen Species Induction by Hepatitis B Virus: Implications for Viral Replication in p53-Positive Human Hepatoma Cells

**DOI:** 10.3390/ijms25126606

**Published:** 2024-06-15

**Authors:** Yuna Jeong, Jiwoo Han, Kyung Lib Jang

**Affiliations:** 1 Department of Integrated Biological Science, The Graduate School, Pusan National University, Busan 46241, Republic of Korea; da3967@naver.com (Y.J.); hanjiwoo@pusan.ac.kr (J.H.); 2 Department of Microbiology, College of Natural Science, Pusan National University, Busan 46241, Republic of Korea; 3 Microbiological Resource Research Institute, Pusan National University, Busan 46241, Republic of Korea

**Keywords:** HBx, Hepatitis B virus, proteasome, reactive oxygen species, Siah-1, p53

## Abstract

Hepatitis B virus (HBV) infects approximately 300 million people worldwide, causing chronic infections. The HBV X protein (HBx) is crucial for viral replication and induces reactive oxygen species (ROS), leading to cellular damage. This study explores the relationship between HBx-induced ROS, p53 activation, and HBV replication. Using HepG2 and Hep3B cell lines that express the HBV receptor NTCP, we compared ROS generation and HBV replication relative to p53 status. Results indicated that HBV infection significantly increased ROS levels in p53-positive HepG2-NTCP cells compared to p53-deficient Hep3B-NTCP cells. Knockdown of p53 reduced ROS levels and enhanced HBV replication in HepG2-NTCP cells, whereas p53 overexpression increased ROS and inhibited HBV replication in Hep3B-NTCP cells. The ROS scavenger N-acetyl-L-cysteine (NAC) reversed these effects. The study also found that ROS-induced degradation of the HBx is mediated by the E3 ligase Siah-1, which is activated by p53. Mutations in p53 or inhibition of its transcriptional activity prevented ROS-mediated HBx degradation and HBV inhibition. These findings reveal a p53-dependent negative feedback loop where HBx-induced ROS increases p53 levels, leading to Siah-1-mediated HBx degradation and HBV replication inhibition. This study offers insights into the molecular mechanisms of HBV replication and identifies potential therapeutic targets involving ROS and p53 pathways.

## 1. Introduction

The infection of the hepatitis B virus (HBV)—a member of the *Hepadnaviridae* family—poses a significant public health challenge, affecting approximately 350 million individuals globally with chronic HBV infection [1]. This condition is associated with a high risk of developing severe liver diseases, including acute and chronic hepatitis, cirrhosis, and hepatocellular carcinoma [1]. The HBV replicates a partially double-stranded circular DNA genome of approximately 3200 base pairs through reverse transcription of its pregenomic RNA (pgRNA) [2]. This genome includes four major open reading frames (ORFs) that encode surface antigens (preS1, preS2, and S proteins), core antigens (preC and C proteins), polymerase (P protein), and X protein (HBx). The HBx is a 17-kDa multifunctional oncoprotein that influences signal transduction, transcription, and mitochondrial function and is found in the cytoplasm, nucleus, and mitochondria [3,4]. The HBx is recognized as a positive regulator of HBV replication, as demonstrated in various models including in vitro infection systems, human hepatocyte chimeric mice, and murine hydrodynamic injection studies [5,6,7]. The HBx activates viral promoters to synthesize HBV mRNA and pgRNA from a covalently closed circular DNA template [2]. Additionally, HBx indirectly promotes HBV replication by disrupting cellular signaling pathways such as the cytosolic calcium signaling pathway and the phosphatidylinositol 3-kinase/Akt pathway [8,9].

Reactive oxygen species (ROS) are highly reactive molecules derived from oxygen. Under normal circumstances, cells generate ROS continuously through processes like protein folding in the endoplasmic reticulum, oxidative phosphorylation in mitochondria, and the catabolism of lipids and amino acids in the cytosol [10]. It has been demonstrated that ROS levels are frequently elevated in the liver and blood of HBV-infected patients [11,12]. Moreover, in vitro HBV infection has been shown to induce ROS production in cells [13]. While viral proteins like the HBsAg and the HBcAg may contribute to this process [14], HBx is considered the primary driver of oxidative stress in the course of HBV infection. For instance, the HBx interacts with the human voltage-dependent anion channel in mitochondria, affecting its transmembrane potential [15,16] and decreasing mitochondrial enzymes involved in electron transport, resulting in elevated ROS levels in mitochondria [17]. Excess ROS from chronic HBV infection can damage proteins, lipids, and DNA, potentially leading to liver fibrosis and hepatocellular carcinoma [18,19]. Although ROS plays a well-established role in HBV pathogenesis, their impact on HBV replication is not yet fully understood.

Several studies have suggested that the HBx levels are primarily regulated by the E3 ligase seven in absentia homolog 1 (Siah-1), which facilitates ubiquitin (Ub)-dependent proteasomal degradation of the HBx. Additionally, p53 can stimulate the transcriptional activation of *Siah-1* via p53 response elements located in its promoter [20]. Given that p53 is a crucial mediator of DNA damage induced by excess ROS [21], it is valuable to investigate whether HBx-induced ROS triggers *Siah-1* expression through p53 activation. This could lead to the downregulation of the HBx via a negative feedback loop, thereby inhibiting HBV replication. For this study, we used an optimized in vitro HBV replication system [22,23] to accurately evaluate the effect of HBx-induced ROS on HBV replication. For this purpose, we first investigated whether the HBV induces varying levels of ROS generation depending on the p53 status in human hepatoma cells. Following this, we examined the mechanism by which the HBV amplifies ROS in a p53-dependent manner. Subsequently, we explored whether HBV-induced ROS negatively regulates HBV replication in hepatoma cells. Furthermore, we assessed the role of the HBx in the p53-dependent ROS amplification and the ROS-mediated inhibition of the HBV in these cells. Finally, we investigated whether ROS upregulates *Siah-1* expression by elevating p53 levels and subsequently reduces HBx levels through Siah-1-mediated proteasomal degradation.

## 2. Results

### 2.1. Exploring the Relationship between ROS Induction and HBV Replication in p53-Positive Human Hepatoma Cells

**Figure 1:**
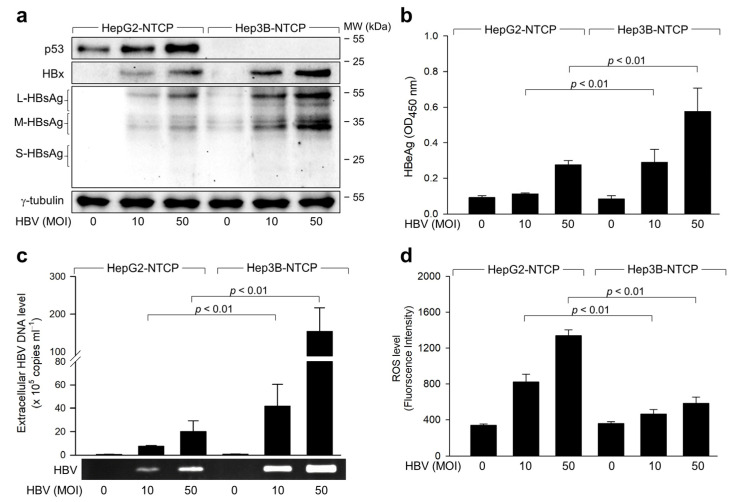
Exploring the relationship between ROS induction and HBV replication in p53-positive human hepatoma cells: (**a**) HepG2-NTCP and Hep3B-NTCP cells were infected with HBV at 10 or 50 MOI per cell for 24 h in DMEM with 2% DMSO and 4% PEG 8000. The cells were washed twice with DMEM without serum and incubated for an additional three days in DMEM with 3% FBS, 2% DMSO, and 4% PEG 8000. Cell lysates were analyzed by western blotting to measure the levels of indicated proteins. (**b**) Levels of HBeAg derived from the cells prepared in (**a**) were detected by ELISA. Results are presented as mean ± standard deviation from five independent experiments (*n* = 5). (**c**) Levels of extracellular HBV DNA from (**a**) were determined using both conventional PCR and quantitative real-time PCR (qPCR) (*n* = 4). (**d**) Intracellular ROS levels were assessed with chloromethyl dichlorodihydrofluorescein diacetate (CM-H2DCFDA; Invitrogen), a widely used H_2_O_2_-specific probe, in intact cells [30]. The conversion of CM-H2DCFDA to the green fluorescent product, DCF, was measured using a microplate reader with excitation and emission wavelengths of 485 nm and 535 nm, respectively (*n* = 4).

Initially, we explored whether HBV infection leads to differential ROS generation in human hepatoma cells based on the p53 status. HepG2 cells express wild-type (WT) p53, whereas Hep3B cells do not, providing a unique platform for comparative studies on the role of p53 in HBV-related molecular mechanisms [24]. For in vitro HBV replication studies, HepG2 and Hep3B cell lines were stably transfected with a plasmid encoding the HBV receptor, sodium taurocholate co-transporting polypeptide (NTCP) [25], to establish HepG2-NTCP and Hep3B-NTCP cell lines, respectively [26]. These cell lines support the entire HBV life cycle, from entry to the release of infectious virions. HBV particles were produced from a 1.2-mer HBV replicon [26,27]. Replication of the HBV in both HepG2-NTCP and Hep3B-NTCP cells was confirmed by western blot analysis of viral proteins such as the HBx and HBsAg in cell lysates (Figure 1a), detection of extracellular hepatitis B envelope antigen (HBeAg) by ELISA (Figure 1b), and quantification of virus particles in cell culture media using conventional PCR and quantitative real-time PCR (qPCR) (Figure 1c). Large amounts of large (L)- and middle (M)-HBsAg, along with trace amounts of small (S)-HBsAg, were detected in the infected cells (Figure 1a), consistent with previous findings [26]. In contrast to HepG2-NTCP cells, small quantities of HBsAg derived from the inserted viral genome [28] were present in the cell lysates of uninfected Hep3B-NTCP cells (Figure 1a, lane 4), while neither conventional PCR nor qPCR detected evidence of HBV replication in these cells (Figure 1c, lane 4). These findings suggest that the HBV replicates successfully in HepG2-NTCP and Hep3B-NTCP cells under our experimental conditions. In line with prior studies that identified p53 as a negative regulator of HBV replication [26,29], all three experimental methods revealed higher levels of HBV replication in Hep3B-NTCP cells (Figure 1a–c).

We next focused on determining whether the HBV induces differential ROS generation based on the p53 status during its replication in human hepatoma cells. In alignment with prior research [31], HBV infection elevated intracellular ROS levels in both cell lines (Figure 1d). Notably, the effect of HBV infection on ROS levels was markedly more pronounced in HepG2-NTCP cells (approximately 4-fold increase) compared to Hep3B-NTCP cells (approximately 1.5-fold increase). These observations align with a previous study [32,33] demonstrating the role of p53 in amplifying ROS levels during HBV infection. Moreover, the inverse relationship between HBV and ROS levels hints at a possible role for ROS in negatively regulating HBV replication2.2. HBV Activates p53 to Amplify ROS during Infection in Human Hepatoma Cells

**Figure 2:**
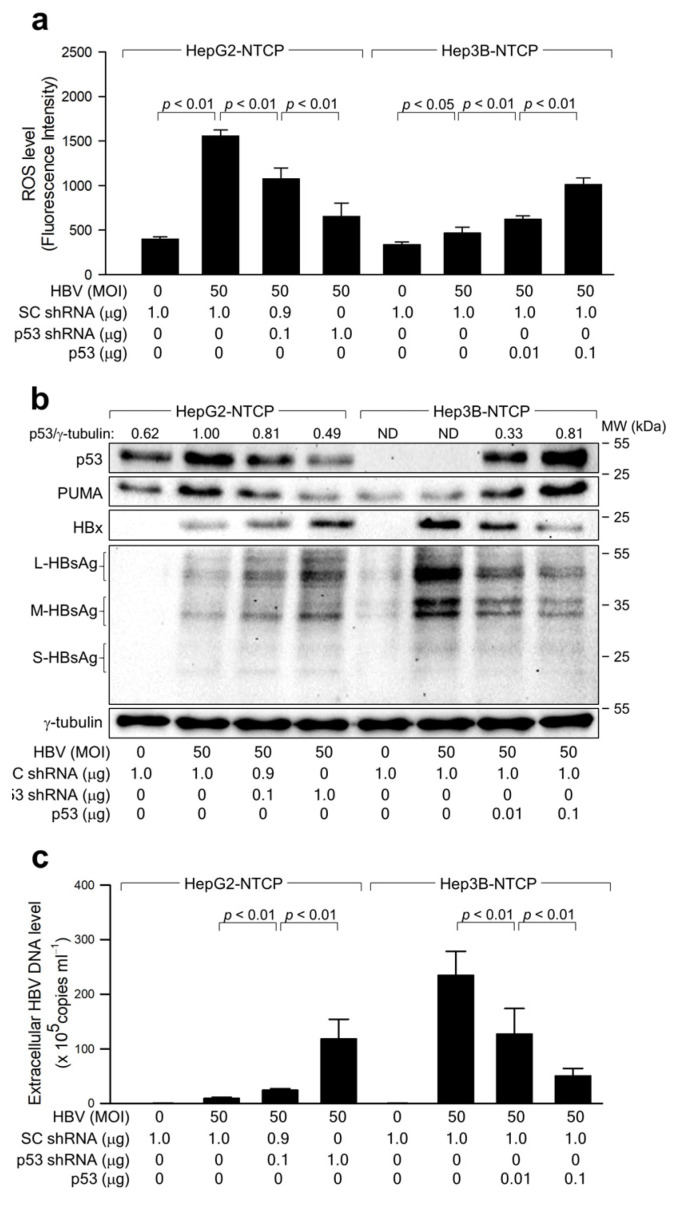
HBV activates p53 to amplify ROS during infection in human hepatoma cells. (**a**) HepG2-NTCP and Hep3B-NTCP cells were transfected with p53 shRNA and p53 expression plasmid for 24 h. Cells were then infected with HBV at 50 MOI for an additional 3 days. Intracellular ROS levels were analyzed as in Figure 1d (*n* = 4). (**b**) The levels of the specified proteins were measured by western blotting. Each protein band’s normalized intensity was calculated relative to the housekeeping protein γ-tubulin to measure protein expression levels across samples. (**c**) Extracellular HBV DNA levels were quantified using qPCR (*n* = 4).

We further investigated the mechanism by which HBV triggers ROS amplification in a p53-dependent manner. In line with earlier findings [20,34], HBV infection led to an upregulation of p53 levels in HepG2-NTCP cells (Figure 1a). To ascertain the role of p53 activation in ROS amplification, we employed p53 shRNA to knock down p53 in HepG2-NTCP cells and used ectopic p53 expression to overexpress p53 in Hep3B-NTCP cells. In this study, scrambled short hairpin RNA (SC shRNA) served as a negative control, ensuring that the effects observed are due to specific gene silencing rather than nonspecific shRNA actions. Concurrently, p53 shRNA was utilized to specifically knock down the p53 gene, enabling the assessment of the role of p53 in cellular processes. The comparison between these two treatments allowed for clear differentiation of specific gene silencing effects from potential off-target effects or general RNA interference toxicity. As a result, reduction in p53 resulted in decreased ROS levels in HBV-infected HepG2-NTCP cells, while increased p53 expression elevated ROS levels in HBV-infected Hep3B-NTCP cells (Figure 2a). This underscores the involvement of p53 in ROS amplification during HBV infection in human hepatoma cells. Conversely, reducing p53 levels boosted both intracellular viral proteins and extracellular HBV virions in HBV-infected HepG2-NTCP cells, while increased p53 expression curtailed HBV replication in Hep3B-NTCP cells (Figure 2b,c). These findings confirm the role of p53 as a negative modulator of HBV replication in hepatocytes [26,29]. Based on these observations, we conclude that p53 activation plays a dual role: it is both implicated in ROS amplification and contributes to the inhibition of HBV replication in p53-positive human hepatoma cells during HBV infection.

### 2.2. HBV Induces ROS Generation to Inhibit Virus Replication via a Negative Feedback Loop

**Figure 3:**
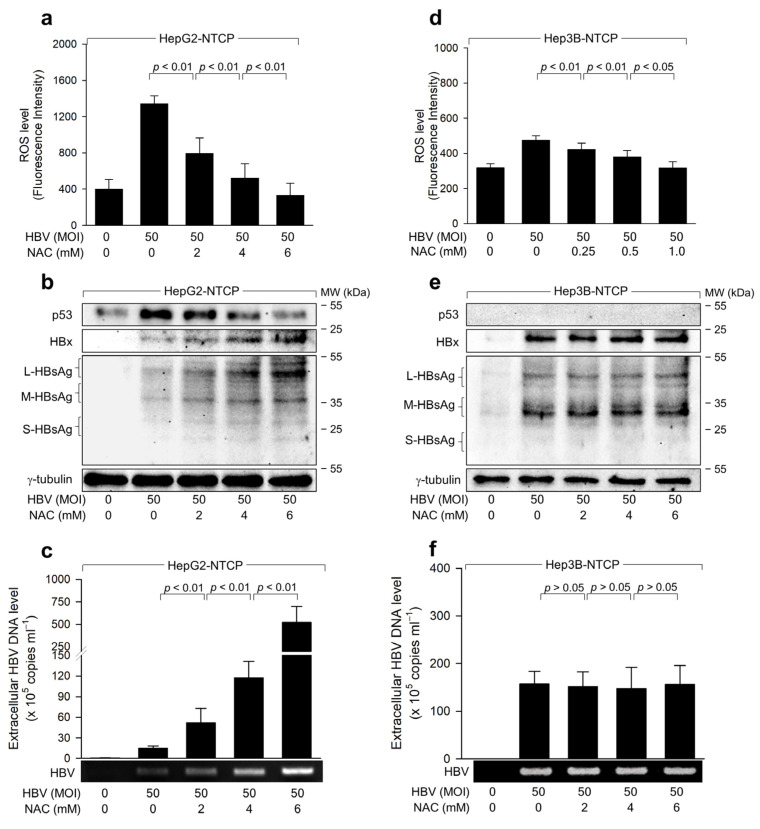
HBV induces ROS generation to inhibit virus replication via a negative feedback loop. (**a**,**d**) HepG2-NTCP and Hep3B-NTCP cells were infected with HBV for 48 h. Cells were then treated with NAC at the specified concentrations for 24 h before harvesting. ROS levels in cells were measured using CM-H2DCFDA (*n* = 5). (**b**,**e**) Levels of p53, HBx, HBsAg, and γ-tubulin in cell lysates were analyzed by western blotting. (**c**,**f**) Extracellular HBV DNA levels were quantified using conventional PCR and qPCR (*n* = 3).

To determine if HBV-induced ROS regulates HBV replication in both cell lines, we assessed the impact of the antioxidant N-acetyl-L-cysteine (NAC) on HBV replication. Known as a potent ROS scavenger, NAC effectively neutralized ROS during HBV infection in a dose-dependent manner, thereby nullifying the ability of the HBV to increase ROS and p53 levels in HepG2-NTCP cells (Figure 3a,b). Furthermore, NAC treatment led to dose-dependent activation of HBV replication in HepG2-NTCP cells, evidenced by the incremental rise in intracellular viral proteins and extracellular virus particles (Figure 3b,c). In Hep3B-NTCP cells, NAC treatment did not affect the levels of intracellular HBV proteins or extracellular HBV virions, although it effectively quelled HBV-induced ROS (Figure 3d–f). From these results, we deduce that HBV-induced ROS acts as an inhibitor of HBV replication in human hepatoma cells, but this inhibition is contingent upon the presence of p53. 

### 2.3. The Transcriptional Activity of p53 Is Crucial for the ROS-Induced Suppression of HBV Replication

**Figure 4:**
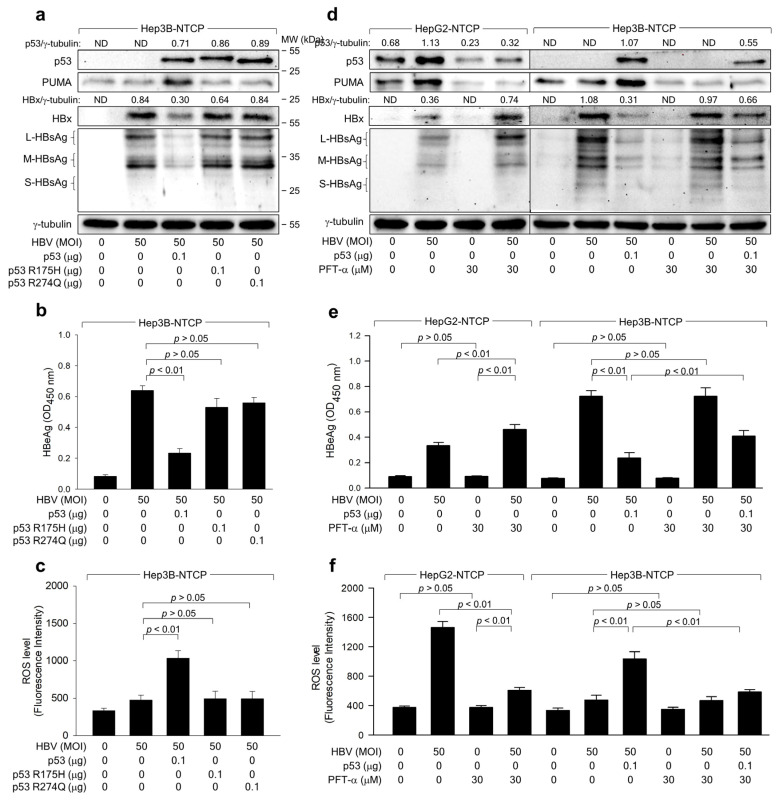
The transcriptional activity of p53 is crucial for the ROS-induced suppression of HBV replication. (**a**–**c**) Hep3B-NTCP cells were transfected with an expression plasmid encoding wild type (WT) p53, p53 R175H, and p53 R248Q for 24 h and then infected with HBV 50 MOI for an additional 3 days. (**d**–**f**) HepG2-NTCP cells and Hep3B-NTCP cells were infected with HBV, followed by treatment with PFT-α at the indicated concentrations. For lanes 7 and 10, Hep3B-NTCP cells were transfected with indicated plasmid before infection. (**a**,**d**) Levels of the intracellular proteins were determined by western blotting. (**b**,**e**) Levels of extracellular HBeAg were determined by ELISA (*n* = 3). (**c**,**f**) Levels of ROS were determined after treatment with CM-H2DCFDA (*n* = 3). (**a**,**d**) Each protein band’s normalized intensity was calculated relative to the housekeeping protein γ-tubulin to measure protein expression levels across samples.

We next explored whether the potential of ROS to inhibit HBV replication depends on p53 transcriptional activity. Two p53 mutants, p53 R175H and p53 R248Q, cannot activate target gene transcription [35]. The protein levels of p53 upregulated modulator of apoptosis (PUMA) were upregulated during HBV infection in Hep3B-NTCP cells in the presence of WT p53, whereas HBV infection either without p53 or with the mutated p53 forms minimally affected PUMA levels (Figure 4a). Under these conditions, WT p53 amplified ROS and suppressed HBV replication in Hep3B-NTCP cells, while neither R175H nor p53 R248Q displayed these effects (Figure 4a–c), suggesting that p53 transcriptional activity is pivotal for ROS amplification and subsequent HBV inhibition in human hepatoma cells. 

To corroborate the importance of p53 transcriptional activity for ROS-induced HBV inhibition, we used the specific p53 inhibitor, pifithrin-α (PFT-α), which curtails the transcriptional activation of p53 target genes [36]. PFT-α treatment diminished PUMA levels in HepG2-NTCP cells, regardless of HBV infection status, thereby nearly abolishing the effect of the HBV on PUMA levels (Figure 4d). This indicates successful inhibition of p53 transcriptional activity by PFT-α during HBV infection in HepG2-NTCP cells. PFT-α also reduced p53 levels in uninfected and HBV-infected HepG2-NTCP cells (Figure 4d), as it interrupted p53 amplification through a positive feedback loop [37]. However, PFT-α could not entirely negate the effect of the HBV on p53 levels (Figure 4d), likely because initial ROS induction and subsequent p53 upregulation during HBV infection are not contingent on p53 transcriptional activity. In HepG2-NTCP cells, PFT-α stimulated HBV replication, as evidenced by heightened intracellular HBx and HBsAg and extracellular HBeAg levels (Figure 4d,e). Moreover, PFT-α reduced ROS levels by approximately one-third during HBV infection in these cells, whereas the effect was minimal in uninfected cells, possibly due to ROS detection limit (Figure 4f). Conversely, PFT-α did not induce noticeable changes in ROS production and HBV replication in Hep3B-NTCP cells (Figure 4d−f). Ectopic p53 expression restored the potential of PFT-α to diminish ROS levels and stimulate HBV replication in Hep3B-NTCP cells (Figure 4d–f). Based on these observations, we conclude that p53 transcriptional activity is indispensable for HBV-induced ROS generation and the subsequent p53-mediated inhibition of HBV replication in human hepatoma cells. 

### 2.4. ROS Reduces HBx Levels to Suppress HBV Replication in Human Hepatoma Cells

**Figure 5:**
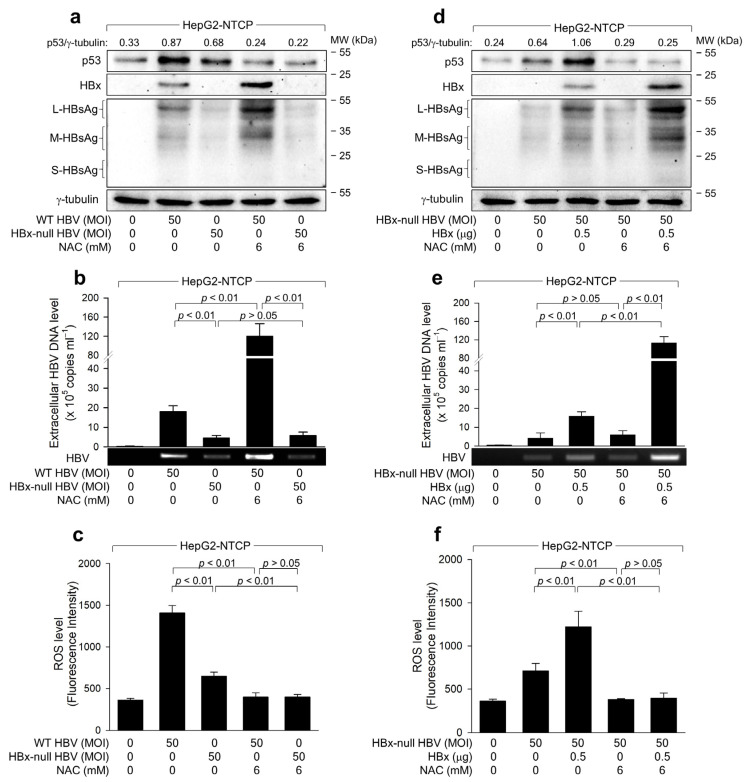
ROS reduces HBx levels to suppress HBV replication in human hepatoma cells. (**a**) HepG2-NTCP cells were infected with either WT HBV or HBx-null HBV for 3 days. If required, cells were treated with NAC for 24 h before harvesting. For lanes 3 and 5 in (**d**–**f**), pCMV-HA-HBx was transfected 24 h before HBV infection. (**a**,**d**) The levels of the indicated proteins were measured by western blotting. (**b**,**e**) Extracellular HBV DNA levels were quantified using conventional PCR and qPCR (*n* = 3). (**c**,**f**) ROS levels were measured after treatment with CM-H2DCFDA (*n* = 3). (**a**,**d**) The normalized intensity of each protein band was calculated relative to the housekeeping protein γ-tubulin to compare protein expression levels across samples.

Next, we examined the potential role of HBx in p53-dependent ROS amplification and ROS-mediated HBV inhibition in hepatoma cells. HepG2-NTCP cells were infected with either WT HBV or its HBx-null variant. Notably, WT HBV displayed a significantly higher replication rate than HBx-null HBV, which is evident from elevated levels of intracellular HBsAg and extracellular virions (Figure 5a,b). The potential of WT HBV to induce ROS and elevate p53 levels during infection in HepG2-NTCP cells was also more pronounced compared to HBx-null HBV (Figure 5a,c). Furthermore, ectopic *HBx* expression largely complemented the defects observed in HBx-null HBV (Figure 5d–f), confirming the pivotal roles of the HBx in HBV replication [6,26,27], ROS generation [13,17], and p53 activation [26]. Intriguingly, HBx-null HBV still managed to induce ROS during replication in HepG2-NTCP cells (Figure 5c), implying potential contributions from other viral factors such as HBsAg and HBcAg in this mechanism. Treatment with NAC significantly attenuated the potential of WT HBV to elevate p53 and ROS levels during infection in HepG2-NTCP cells (Figure 5a,c). Likewise, NAC abolished the potential of HBx-null HBV to augment ROS and p53 levels in HepG2-NTCP cells with *HBx* expression (Figure 5d,f). Furthermore, NAC promoted the replication of HBx-null HBV in the presence of the HBx and WT HBV in HepG2-NTCP cells (Figure 5a,b,d,e). Conversely, in the absence of the HBx, NAC had a marginal effect on HBx-null HBV replication, even though it effectively neutralized the virus-induced alterations of p53 and ROS levels (Figure 5a–c). These findings suggest that HBx predominantly mediates the p53-dependent inhibition of HBV replication provoked by ROS. 

### 2.5. ROS Drives Siah-1-Mediated Proteasomal Degradation of HBx in a p53-Reliant Manner

**Figure 6:**
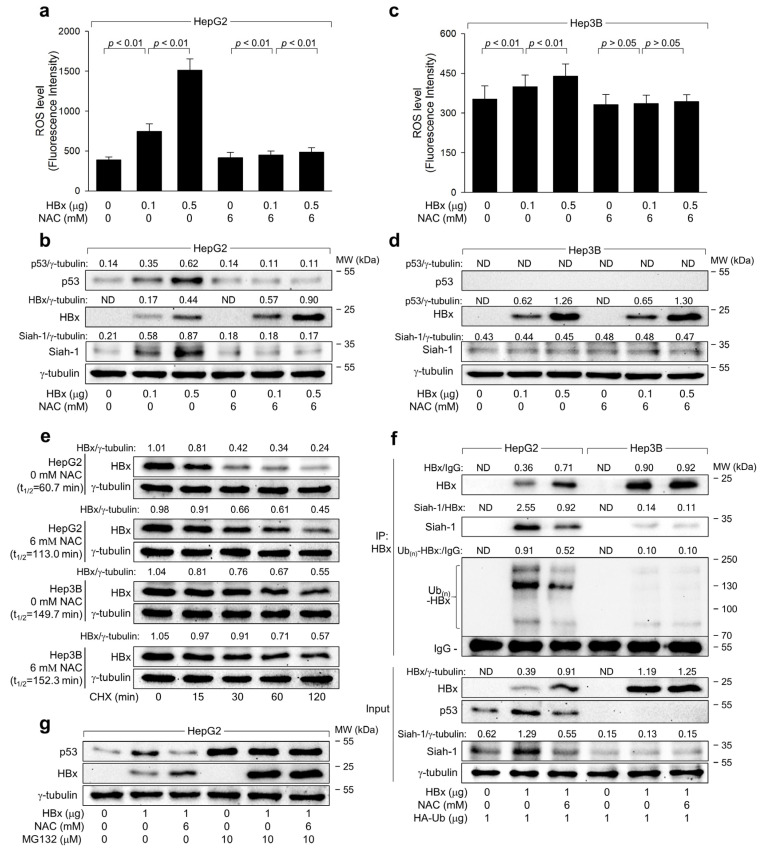
ROS drives Siah-1-mediated proteasomal degradation of HBx in a p53-reliant manner. HepG2 and Hep3B cells were transfected with an *HBx* expression plasmid for 48 h in the presence or absence of NAC. (**a**,**c**) ROS levels were determined (*n* = 4). (**b**,**d**) Levels of the indicated proteins were determined by western blotting. (**e**) Cells were treated with 50 μg/mL of cycloheximide (CHX) for the indicated time to inhibit further protein synthesis before harvesting, followed by western blotting. Each band was quantified using ImageJ image-analysis software (Version 2.1.0, NIH) to determine the half-life (t_1/2_) of HBx protein (**f**) HA-Ub expression plasmid was added in the transfection cocktails. Ubiquitin-conjugated HBx products were immunoprecipitated with anti-HA antibodies and pulled down with magnetic beads. Western blotting was performed to measure levels of HBx, p53, Siah-1, and Ub-complexed HBx. The input lanes show the levels of the proteins. (**g**) Cells were mock-treated or treated with 10 μM MG132 for 4 h before protein sampling. (**b**,**d**,**e**,**f**) Each protein band’s normalized intensity was calculated relative to the housekeeping protein γ-tubulin to measure protein expression levels across samples.

To elucidate the direct roles of the HBx in ROS generation and subsequent p53 activation, we transiently transfected HBx in HepG2 and Hep3B cells. Ectopic *HBx* expression alone elevated ROS and p53 levels in HepG2 cells (Figure 6a,b). In Hep3B cells, although the HBx slightly upregulated ROS levels, the effect was less pronounced than in HepG2 cells (Figure 6c,d). This suggests that HBx initially induces ROS generation via a p53-independent mechanism, which is further amplified mainly through p53 upregulation, consistent with a previous study [32]. Notably, p53 knockdown resulted in elevated HBx levels during HBV infection in HepG2-NTCP cells, whereas p53 overexpression led to reduced HBx levels in Hep3B-NTCP cells (Figure 2b), aligning with previous findings on the negative regulatory effect of p53 on the HBx during HBV infection [20,26,29,38]. Moreover, ROS depletion using NAC increased HBx levels during HBV infection and ectopic expression in HepG2-NTCP cells (Figure 5a and Figure 6b). However, NAC had minimal effect on HBx levels during HBV replication and ectopic expression in Hep3B-NTCP cells where no p53 signal was detected (Figure 3e and Figure 6d). These data strongly link HBx-induced ROS generation to p53-mediated HBx downregulation in hepatoma cells. 

Previous reports indicate that p53 transcriptionally activates the *Siah-1* gene, which encodes an E3 ligase for the HBx [20,23,39]. Indeed, ectopic *HBx* expression, independent of other viral proteins, upregulated Siah-1 levels via p53 upregulation in HepG2 cells but not in Hep3B cells (Figure 6b,d). Additionally, NAC treatment reduced p53 and Siah-1 levels in HepG2 but not in Hep3B cells (Figure 6b,d). These findings strongly suggest that Siah-1 is involved in ROS-mediated HBx downregulation in a p53-dependent manner. To substantiate that ROS reduces HBx levels via p53-dependent Siah-1 upregulation, we evaluated if ROS affects HBx protein stability in a p53-dependent manner. We treated HBx-expressing HepG2 and Hep3B cells with or without NAC, followed by cycloheximide (CHX) treatment to halt protein synthesis. The half-life (t_1/2_) of the HBx in HepG2 cells was 60.7 min and increased to 113.0 min with NAC, while in Hep3B cells, it was 149.7 min and minimally affected by NAC (Figure 6e), confirming the role of ROS in HBx destabilization via p53.

Additionally, we investigated whether ROS enhances Siah-1-mediated ubiquitination of the HBx through p53 dependence. We co-transfected *HBx* and HA-tagged ubiquitin into both cell lines, with or without NAC treatment, and immunoprecipitated the ubiquitin-complexed HBx. Co-immunoprecipitation revealed that Siah-1 interacted with the HBx, inducing its ubiquitination in HepG2 cells (Figure 6f, lane 2). In contrast, this interaction and subsequent HBx ubiquitination were compromised in Hep3B cells due to the absence of p53-mediated Siah-1 upregulation (Figure 6f, lane 5). NAC treatment reduced HBx-Siah-1 interaction in HepG2 cells, decreasing HBx ubiquitination and elevating its protein levels, whereas these effects were largely absent in Hep3B cells (Figure 6f, lanes 3 and 6). MG132, a proteasomal inhibitor, attenuated HBx and NAC-induced p53 and HBx upregulation in HepG2 cells (Figure 6g). In conclusion, these findings establish that Siah-1 drives ROS-induced ubiquitination and proteasomal degradation of the HBx in a p53-dependent manner. 

## 3. Discussion

HBV infection typically induces oxidative stress, marked by increased levels of ROS, such as H_2_O_2_, in the liver and blood of patients, and both host and viral factors are known to be included in this process [11,12]. Virus-specific cytotoxic T lymphocytes, which are mainly responsible for liver injury by destroying infected hepatocytes and promoting the production of inflammatory cytokines, significantly contribute to ROS generation in the liver [40]. Additionally, the HBV contributes to the production of ROS in hepatocytes, and this effect is primarily mediated by the HBx [13,14,15,17]. In this study, we employed an optimized HBV infection system, which allows successful HBV infection [22]. Infection of HepG2-NTCP cells with the HBV at an indicated multiplicity of infection (MOI) for 4 days enabled quantitative analysis of HBV replication rates through measurement of HBV proteins in the cell lysates and extracellular HBV particles in the culture media (Figure 1). Our findings revealed a significant difference in ROS levels between HepG2 and Hep3B cells, suggesting a potential role for p53 in ROS modulation. Given the inherent limitations of using Hep3B cells, including the possible impact of genomic integration, we further sought to clarify the role of p53 in regulating ROS. To this end, we manipulated p53 expression by knocking down its expression in HepG2 cells and ectopically expressing it in Hep3B cells (Figure 2a). These manipulations consistently demonstrated that ROS levels are influenced by the status of p53, thereby reinforcing the link between p53 and ROS regulation. To conclusively determine the influence of p53 on ROS and HBV replication, we employed p53 inhibitors, specifically the p53 dominant negative mutant R175H and R248Q, and PFT-α, to disrupt normal p53 function. The application of these inhibitors resulted in the attenuation of the p53 effects on ROS and subsequent HBV replication (Figure 4), thereby confirming the interdependence of p53, ROS production, and HBV replication dynamics. These results collectively highlight the critical role of p53 in modulating ROS levels and its subsequent impact on HBV replication, providing valuable insights into the cellular mechanisms governing viral replication in liver cells.

The HBx appears to play a predominant role in ROS production during HBV infection. When localized within mitochondria, the HBx alters mitochondrial membrane potential, triggering ROS generation [15,16]. Additionally, the HBx modulates the permeability of the mitochondrial membrane and disrupts the mitochondrial respiratory chain [15,41]. HBx-induced ROS may induce DNA damage, such as double-strand DNA breaks, resulting in activation of the ATM-Chk2 pathway and subsequent stabilization of p53. Previous reports have demonstrated that the HBx induces the accumulation of γ-H2AX [42] and activates ATM and Chk2 via phosphorylation at the Ser-1981 and Thr-68 residues, respectively, leading to activation of p53 via phosphorylation at the Ser-15 and Ser-20 residues. Consistently, the present study showed that ectopic *HBx* expression alone is sufficient to elevate both ROS and p53 levels in hepatoma cells (Figure 6). While HBx-null HBV also induces ROS generation and activates p53 (Figure 5a), presumably due to the possible roles of HBsAg and HBcAg [43], the magnitude of these effects appears considerably weaker compared to that observed in the presence of the HBx (Figure 1 and Figure 6).

The slight increase in ROS levels in Hep3B-NTCP cells following HBV infection (Figure 1) suggests that HBV can trigger ROS production through a p53-independent mechanism. The involvement of the HBx in this phenomenon was validated through ectopic expression experiments in Hep3B cells, where the HBx alone induced ROS elevation independent of other viral proteins (Figure 6c). This effect was likely due to the potential of the HBx to induce ROS generation in mitochondria through p53-independent mechanisms [15,16,41]. However, the ROS levels generated by the HBx in the absence of p53 were considerably lower than those obtained in the presence of p53 (Figure 6a,c), underscoring the crucial role of p53 in amplifying ROS in HBV-infected cells. Many p53-inducible genes encode redox-active proteins involved in ROS generation, such as quinone oxidoreductase, proline oxidase, and manganese superoxide dismutase [44,45]. Therefore, p53 may amplify its protein levels through a positive feedback loop involving ROS and the ATM-ChK2 pathway.

The R175 residue located within the zinc-binding site adjacent to the DNA-binding interface of p53 is crucial for maintaining structural stability, while R248 is involved in direct interaction with DNA [35]. Mutations in these residues result in the loss of transcriptional activator function and interfere with p53 tetramer formation as dominant negatives. Consistently, both *p53 R175H* and *p53 R248Q* expressed in Hep3B-NTCP cells did not exhibit transcriptional activity, as demonstrated by the protein levels of PUMA (Figure 4a). Neither mutation induced ROS amplification nor inhibited HBV replication (Figure 4a–c), indicating the importance of p53 transcriptional activity in regulating ROS production and HBV replication. Additionally, treatment with PFT-α, which decreases nuclear p53 stability [46] or its DNA binding activity [47], stimulated HBV replication but decreased ROS levels in both HepG2-NTCP and Hep3B-NTCP cells (Figure 4d–f). This suggests that transcriptional activity is required for p53 to regulate ROS generation and viral replication. Interestingly, the potential of p53 to inhibit HBV replication remained active even when treatment with PFT-α completely inhibited the ability of p53 to activate the expression of its target genes. This suggests that p53 can function as a negative regulator of HBV replication through another mechanism that does not involve its transcriptional activity.

The role of p53 as a negative regulator of HBV replication is relatively well-established [26,32,48]. The present study provides several lines of evidence supporting that HBx-induced ROS inhibits HBV replication by downregulating HBx levels in a p53-dependent manner. First, lower levels of intracellular HBx and HBsAg and extracellular HBeAg and HBV particles were produced during HBV infection in HepG2-NTCP cells where the HBx effectively upregulated p53 and ROS levels (Figure 1). Second, p53 knockdown downregulated ROS levels but stimulated HBV replication in HepG2-NTCP cells, whereas ectopic p53 expression increased ROS levels but inhibited HBV replication in Hep3B-NTCP cells (Figure 2). Third, HBV replication was enhanced when ROS levels were lowered by treatment with NAC (Figure 3a–c), as demonstrated in a previous study suggesting the negative role of ROS in HBV replication [49]. Notably, this effect was not observed during WT HBV infection in Hep3B-NTCP cells and HBx-null HBV infection in HepG2-NTCP and Hep3B NTCP cells, where p53 was largely unaffected or absent (Figure 3 and Figure 5), indicating the significant role of p53 activation in this process.

It has been shown that p53 reduces HBx levels through ubiquitin-dependent proteasomal degradation, inhibiting HBV replication [20,50]. Siah-1, acting as an E3 ligase for the HBx, facilitates the ability of p53 to downregulate HBx levels. [20,39]. Given that *Siah-1* expression is recognized to be transcriptionally activated by p53 [20,51], it is conceivable that the p53 activated by HBx-induced ROS could stimulate the expression of *Siah-1*. Consistently, ectopic *HBx* expression upregulated both p53 and Siah-1 levels in HepG2 cells, whereas treatment with NAC nullified the ability of the HBx to induce this effect (Figure 6b). In addition, treatment with NAC decreased the binding of Siah-1 to the HBx and HBx ubiquitination, resulting in the upregulation of HBx levels in a p53-dependent manner (Figure 6). Given the potential of the HBx to enhance HBV replication [6,52,53], as also shown in the current study (Figure 5), the Siah-1-mediated ubiquitination and proteasomal degradation of the HBx likely play a crucial role in ROS-induced inhibition of HBV replication.

This study demonstrated that the HBx potentially contributes to ROS-induced inhibition of HBV replication by being downregulated through a negative feedback loop. The reasons why the HBx limits its expression during HBV replication via this feedback loop involving ROS and p53 are not yet clear. One possibility is that the HBV modulates HBx protein levels to remain within a specific threshold during persistent infection. Alternatively, the ROS-induced inhibition of HBV replication may serve as an innate host defense mechanism against HBV infection, highlighting p53’s role as the guardian of the genome. By demonstrating the inhibitory effect of ROS on HBV replication in a p53-dependent manner and elucidating the involvement of Siah-1 in HBx degradation, this research underscores the therapeutic potential of targeting ROS signaling pathways for HBV treatment. Further studies on ROS-inducing agents could pave the way for innovative strategies to combat HBV and enhance patient outcomes. A limitation of the current study is that it does not address the long-term effects of chronic infection, as the current in vitro system is not suitable for studying prolonged infection periods necessary to confirm chronic infection. To address the role of p53 in ROS generation and HBV replication during chronic hepatitis B, in vivo experiments based on the p53 status of patients should be performed.

## 4. Materials and Methods

### 4.1. Plasmid

The plasmid pCMV-3 × HA1-HBx (HA-HBx) carries the *HBx* gene (genotype D) situated downstream from three influenza virus haemagglutinin (HA) tags [54]. The 1.2-mer WT HBV replicon, which includes 1.2 copies of the genotype D HBV genome, and a version without the *HBx* gene were provided by W. S. Ryu (Yonsei University, Seoul, Republic of Korea) [27]. The plasmid RC210241, which codes for the human *NTCP*, was sourced from OriGene (Rockville, MD, USA, Cat No. 003049). Scrambled (SC) and p53-targeting short hairpin RNA (shRNA) were acquired from Santa Cruz Biotechnology (Santa Cruz, CA, USA, Cat No. sc-37007 and sc-29435, respectively). The pCMV p53-WT plasmid was a gift from Chang-Woo Lee (Sungkyunkwan University, Suwon, Republic of Korea). The pCMV p53 R175H and pCMV p53 R274Q plasmids were provided by Bum-Joon Park (Pusan National University, Busan, Republic of Korea). The plasmid pHA-Ub that encodes HA-tagged ubiquitin was generously supplied by Y. Xiong (University of North Carolina at Chapel Hill, NC, USA).

### 4.2. Cell Culture and Transfection

The HepG2 (Cat No. 88065) and Hep3B (Cat No. 88064) cell lines were gained from the Korean Cell Line Bank (KCLB, Seoul, Republic of Korea). To create stable cell lines, HepG2-NTCP and Hep3B-NTCP cells were transfected with *NTCP* expression plasmid and then selected using 500 µg/mL G418 sulfate (Sigma-Aldrich, St. Louis, MO, USA, Cat No. A1720). These cells were cultured in Dulbecco’s Modified Eagle Medium (DMEM) by WelGENE (Gyeongsan, Republic of Korea, Cat No. LM001-05), with 10% fetal bovine serum (FBS, Capricorn Scientific, Ebsdorfergrund, Germany, Cat No. FBS-22A), 100 µg/mL streptomycin (United States Biological, Salem, MA, USA, Cat No. 21865), and 100 units/mL penicillin G (Sigma-Aldrich, Cat No. P3032). The culture conditions were maintained at 37 °C in a 5% CO_2_-humidified atmosphere. For transient expression studies, 2 × 10^5^ cells per well in a 6-well plate were transfected with a transfection reagent (Thermo Fisher Scientific, Waltham, MA, USA, Cat No. R0532). The cells were subjected to various treatments, including CHX(Sigma-Aldrich, Cat No. C7698), N-acetylcysteine (NAC, Sigma-Aldrich, Cat No. A7250), (PFT-α, Sigma-Aldrich, Cat No. P4359), and MG132 (Millipore, Burlington, MA, USA, Cat No. 474790), under specific experimental conditions.

### 4.3. HBV Cell Culture System

For HBV stock preparations, Hep3B-NTCP cells were transfected with the 1.2-mer HBV replicon plasmid as described above. The culture medium was collected to prepare the HBV stocks. The concentration of HBV in these stocks was assessed by qPCR, details of which are provided in the following section. For the infection experiments, HBV was introduced into cells seeded in 6-well plates at a MOI of 50, based on a slightly modified protocol from an established HBV cell culture protocol [22,23]. Initially, 2 × 10^5^ cells per well were exposed to 1 × 10^6^ genome equivalents (GEQs) of HBV and incubated for 24 h in serum-free DMEM supplemented with 2% DMSO (Sigma-Aldrich, Cat No. D8418) and 4% PEG 8000 (Sigma-Aldrich, Cat No. D4463). After two PBS washes, the cells were further cultured in DMEM enriched with 3% FBS, 4% PEG 8000, and 2% DMSO for 72 h.

### 4.4. Quantitative Real-Time PCR of HBV DNA

The extracellular HBV levels were measured using a qPCR protocol [26]. Initially, extracellular HBV DNA was extracted from the collected culture supernatant employing the QIAamp DNA Mini Kit (Qiagen, Hilden, Germany, Cat No. 51306). For conventional PCR analysis, the genomic DNA underwent amplification using 2 × Taq PCR Master Mix 1 (BioFACT, Daejeon, Republic of Korea, Cat No. ST301-19h) with the primers HBV 1399F (5′-TGG TAC CTG CGC GGG ACG TCC TT-3′) and HBV 1632R (5′-AGC TAG CGT TCA CGG TGG TCT CC-3′). Subsequent qPCR analysis was performed using SYBR premix Ex Taq II (Takara Bio, Kusatsu, Japan, Cat No. RR82LR) and the primers HBV 379F (5′-GTG TCT GCG GCG TTT TAT CA-3′) and HBV 476R (5′-GAC AAA CGG GCA ACA TAC CTT-3′) on a Rotor-Gene qPCR machine (Qiagen).

### 4.5. Measurement of Intracellular ROS Levels

Intracellular ROS levels were determined using the H_2_O_2_-specific probe, CM-H2DCFDA(Invitrogen, Waltham, MA, USA, Cat No. C6827), which is commonly used for detecting ROS in living cells [30]. In the procedure, 1 × 10^5^ cells per well were incubated in 12-well plates with 10 µM CM-H2DCFDA in serum-free medium for 30 min. Cells were washed with PBS and collected using Trypsin-EDTA (Gibco, Cleveland, TN, USA, Cat No. 25200-072) for analysis. The fluorescence intensity of DCF was analyzed using a fluorescence microplate reader (Mithras LB940, Berthold Technologies, Bad Wildbad, Germany) set to excitation and emission wavelengths of 485 nm and 535 nm, respectively.

### 4.6. Western Blot Analysis 

Cells were ruptured using a lysis buffer composed of 0.1% SDS, 50 mM Tris-HCl (pH 8.0), 1% NP-40, and 150 mM NaCl, which also contained protease inhibitors from Roche (Basel, Switzerland, Cat No. 11836153001). The protein levels in the lysates were determined with a Bio-Rad protein assay kit (Hercules, CA, USA, Cat No. 5000006). Proteins were separated based on sizes by SDS-PAGE. Transferred proteins on a nitrocellulose membrane (Amersham, UK, Cat No. 10600003) were probed with primary antibodies such as Siah-1 (Abcam, Cambridge, UK, Cat No. ab2237, 1:2000), p53 (Santa Cruz Biotechnology, Cat No. sc-126, 1:1000), HBsAg (Santa Cruz Biotechnology, Cat No. sc-53300, 1:400), γ-tubulin (Santa Cruz Biotechnology, Cat No. sc-17787, 1:500), PUMA (Cell signaling, 4976S, 1:2000), HBx (Millipore, Cat No. MAB8419, 1:500), and HA (Santa Cruz Biotechnology, Cat No. sc-7392, 1:500). These were then incubated with HRP-conjugated secondary antibodies, specifically anti-mouse (Bio-Rad, Cat No. BR170-6516, 1:3000), anti-rabbit IgG (H + L)-HRP (Bio-Rad, Cat No. BR170-6515, 1:3000), or anti-goat IgG (H + L)-HRP (Thermo Scientific, Cat No. 31400, 1:10,000). Protein detection was performed using an ECL kit (Advansta, San Jose, CA, USA, Cat No. K-12043-D20) and imaged with the ChemiDoc XRS system (Bio-Rad).

### 4.7. Immunoprecipitation

Immunoprecipitation (IP) assay was performed using a kit from Thermo Fisher Scientific (Classic Magnetic IP/Co-IP, Cat No. 88804), following the manufacturer’s instructions. Initially, 4 × 10^5^ cells were seeded per 60 mm-diameter dish and transfected with designated expression plasmids for 48 h as specified. Cell lysates were then treated with an anti-HBx antibody (Millipore, Cat No. 8419) at 4 °C overnight to facilitate immune complex formation. To isolate the immune complexes, the mixture was incubated with protein A/G beads, which bind to the Fc region of the antibody, allowing the antibody-protein complexes to be precipitated out of the lysate. A magnetic stand (Pierce, Waltham, MA, USA) was used to elute the protein complexes from the beads. The eluted proteins are analyzed by SDS-PAGE, followed by western blotting to detect the presence of the target protein and any interacting partners.

### 4.8. Quantification of Western Blot Images

Western blot images were captured using a standardized protocol to ensure consistent lighting and exposure across all samples [55]. Band intensity quantification was performed using ImageJ software (version 2.1.0, National Institutes of Health, Bethesda, MD, USA). Each band of interest was outlined with a uniformly sized rectangular selection tool to measure the integrated density, which includes the area and mean gray value of the selected region. Background intensity was determined by measuring a similarly sized area adjacent to the bands. This background value was subtracted from the corresponding band intensity to correct for ambient noise and nonspecific signals. The normalized intensity of each protein band was then calculated relative to the intensity of the housekeeping protein γ-tubulin, providing a relative value of protein expression level in each sample. 

### 4.9. Statistical Analysis

The reported data represent the average values and standard deviations calculated from a minimum of three separate experiments. A two-tailed Student’s *t*-test was performed using SigmaPlot (ver 12.5). Statistical significance was determined based on the P values; specifically, a *p* value of 0.05 or less indicates significant differences between experimental groups. Conversely, a *p* value greater than 0.05 was regarded as not statistically significant, suggesting no meaningful difference between the groups under comparison. This statistical method helps to ensure that the observed effects are not due to random variation, providing a reliable measure of the reproducibility and accuracy of the experimental results. 

## Data Availability

The data presented in this study are available from the corresponding author upon reasonable request.
